# The Diagnosis of Nervous and Mental Diseases

**Published:** 1902-07

**Authors:** 


The Diagnosis of Nervous and Mental Diseases By Howell T. Pershing, M. Sc., M. D., Professor of Nervous and Mental Diseases in the University of Denver; Neurologist to St. Luke's Hospital, etc. Small octavo, illustrated. Philadelphia: P. Blakiston's Son & Co. 1901. [Price, $1.25.]

The object of this book is to facilitate the recognition of nervous and mental diseases by physicians who are not specialists in neurology. It does not claim to take the place of a textbook—but serves to make a textbook more serviceable. The author has followed the plan adopted by botanists and other scientists in classifying according to definite morphological conditions and has evolved a very clever and useful scheme. This could hardly be applied to another branch of medicine except neurology, and here it is most applicable. The contents include, besides the recognition of special diseases by the tables, the examination of the patient and the general significance of symptoms; the recognition of organic diseases; the principles of localisation; the signs of hysteria; the diagnosis of neurasthenia and mixed forms of disease. It is an admirable, ingenious production. W.C.K.

